# The Watery Discharges of Pregnant Women

**Published:** 1886-11

**Authors:** C. C. Frederick

**Affiliations:** Lecturer on Obstetrics, Niagara University


### *THE WATERY DISCHARGES OF PREGNANT WOMEN.

By C. C. FREDERICK, M. D.,

Lecturer on Obstetrics, Niagara University.

*Mr. President and Gentlemen*:

To the physician who sees a considerable of obstetric practice it is not so very rare an occurrence to receive word from some one of his patients that her waters have broken. It has occurred to me and I have known of several others. On questioning the

*Read before the Buffalo Obstetrical Society.

messenger or patient the doctor finds that she has not had the usual first stage pain before the watery discharge, and on examination the cervix is found undilated, as it may be weeks before her expected confinement. She may, or may not, have had pain, but not labor pains. I have selected this case to bring before you for discussion to-night, not so much for its theoretical as for its practical consideration. The etiology and pathology we will touch upon, but the diagnosis and the prognosis to mother and child, together with the treatment of the condition, are more essential to us to-night.

Considering the history of the case we may very appropriately ask several questions, most important of which are:

1st. Whence comes this water?

2d. What does it portend?

3d. What shall we do?

The diagnosis involves the differentiation between (1) a discharge of amniotic fluid, or (2) the ammo-chorial water, which sometimes collects between the amnion and chorion, or (3) a hydrorrhœa gravidarum.

The cases of early rupture of the amniotic sac, we all know to be from violence, or hydramnios; the amnio-chorial waters may collect in large quantities, so as to simulate hydramnios, but their escape is followed by a sense of relief to the woman. A discharge from this source can only occur once, and is seldom followed by premature labor. The fluid of hydrorrhœa gravidarum, or endomitritis decidua catarrhalis, as the name implies, results from a catarrhal inflammation of the decidua. Any antecedent endometritis, be it of gonorrhœal, syphilitic, or of other origin will be a starting point, and in a condition of hydræmia the disease would be most sure to develop. The inflammation effects the decidua vera by preference, but may also effect the decidua reflexa. The fluid from the glands of the tract involved is thin, muco-purulent, watery or sero-sanguinolent, and very nearly resembles the amniotic fluid in odor and color.

If free exit be given it, the quantity is small and continuous. If there be any obstacle to its exit, as the normal adhesion between the decidua reflexa and vera or impenetrability of the os from any cause, the secretion will accumulate in considerable quantities and will force its way through or between the decidua and run from the patient with a gush. This discharge may begin as early in pregnancy as the third month, but the more abundant discharges occur in the later months.

It will be seen, therefore, that there can be but one discharge of amniotic fluid, or amnio-chorial waters, while the discharges of hydrorrhœa gravidarum may recur many times during a pregnancy and not cause premature labor. Ordinarily a rupture of the amniotic sac is followed by labor, and some authors go so far as to say it is always followed by labor. In refutation of the latter statement, Dr. Byford, of Chicago, reports a case occurring in the practice of Dr. C. R. Parke, of Illinois, "in which, a discharge of the liquor amnii took place, labor pains came on and the umbilical cord became prolapsed. He replaced the cord and gave ergot. As labor did not progress he finally gave morphine and quieted the pains. In three months the woman was delivered of a living child; mother and child did well."

Now, Mr. President and gentlemen, can there be an absolute differential diagnosis made between these three different conditions? The premature rupture of the membranes, the discharge of the amnio-chorial waters, or the discharges occurring in hydrorrhœa gravidarum.

When a watery discharge comes on synchronously with a fall, or some other violence, we have presumptive evidence that the water comes from the amniotic sac and that it will probably be followed by labor. Rupture of the amniotic sac seldom occurs in the first five months of pregnancy, except by violence or in abortion, as the direct result of uterine contraction.

A rupture of the amnion seldom occurs in the later months of pregnancy except when there is a great excess of amniotic fluid, hydramnios, or by violence. Hydramnios occurs most frequently in older multiparæ, and ordinarily may be suspected to exist by reason of the size of the uterine tumor being out of all proportions to the period of utero-gestation. Here also we have to diagnose differentially between a twin pregnancy, hydratidiform mole and hydramnios. Twin pregnancies can be diagnosed by auscultation and palpation. Hydratidiform moles usually are expelled before the sixth month, and if they remain after that period will be diagnosed by the absence of the fœtal heart, the currant-juice discharge or vesicles from the growth. The discharge of the amnio-chorial waters most uniformly occurs during the later months of pregnancy and cannot be diagnosticated from the amniotic fluid except by examination. If the os be patulous enough to allow the examining finger to be pushed up and the membranes found intact, by making pressure over the fundus with the other hand, as in palpating a cystic tumor. The discharges of hydrorrhœa gravidarum begin early in pregnancy and increase in amount, and may occur frequently every few days or weeks. The second occurrence of a discharge is presumptive evidence of its existence. Then, gentlemen, the question above propounded may be answered thus: There is no absolute differential diagnosis between all these conditions, but by the careful examination and analysis of each case, a diagnosis and prognosis can be formed that is reasonably free from doubt.

What plan of treatment is applicable to each of these conditions? With the amniotic sac ruptured, labor, not far hence, is imminent, and what matters it if the child be past the viable age? If it be before the age of viability, the indicated treatment is rest and anodynes.

With the hope that the water does not come from the amnion, but that it is from one of the other two sources, it is well to place the woman in recumbency and give viburnum and opium; and this is probably the best uniform treatment for each and all of those discharges, that may or may not be accompanied by symptoms of threatened miscarriage or abortion.

The repeated discharge of so much fluid in hydrorrhœa gravidarum is a large drain upon a woman, and needs to be met by active tonic treatment during its continuance.

Are we justified in any manner to take it for granted that a discharge of water from the vagina of a pregnant woman means labor, and if the cervix is undilated, and there are no labor pains, to proceed at once to give ergot and dilate? (I have heard of doctors being censured for failing to do this, after the delivery of a still-born babe). Emphatically, no.

